# Characteristics of Ethical Leadership: Themes Identification Through Convergent Parallel Mixed Method Design From the Pakistan Context

**DOI:** 10.3389/fpsyg.2021.787796

**Published:** 2021-12-17

**Authors:** Hina Shahab, Hafsah Zahur, Naveed Akhtar, Sobia Rashid

**Affiliations:** Department of Management Sciences, National University of Modern Languages, Islamabad, Pakistan

**Keywords:** ethical leadership themes, data triangulation, open-ended questionnaire, convergent parallel track design, simultaneous bi-directional data integration

## Abstract

The current approaches in identifying the characteristics of ethical leadership proceed mainly from a Western perspective based on virtue-driven moral philosophy (i.e., relativism) and frequently ignoring the Asian perspective of morality based on idealism. This study aimed to conduct parallel analysis in convergent design by using qualitative and quantitative methods to extract person-driven ethical leadership themes by considering the Asian context. Using the hypothetico-deductive method, 13 themes were extracted altogether, out of which 4 are new context-driven themes (i.e., altruism, encouragement, collective good, and spiritual transcendence as the emerging themes of ethical leadership in the Asian context).

## Introduction

In recent literature, a considerable debate is going on to define the approaches of ethical leadership. There are two schools of thought that includes relativism and absolutism perspective of ethical leadership. Most of the researchers rely on a relativism-based Western perspective derived through a pragmatic approach. Realism is the reality about the world around us, and we need to deconstruct the learned philosophy and reconstruct it according to our cultural realities. In contrast, the absolutism (realism) based Eastern approach derived ethics from religious and social-based values that have been completely ignored in the ethical leadership literature (Brown et al., 2005; Alzola et al., 2020; Anser et al., 2021). Ethical leaders’ norms and expectations in Eastern societies are different from the Western perspective, as they are rooted in the spiritual aspects of everyday life. Therefore, it is suggested that the global view of ethical leadership derived from specific cultural values and practices should be explored (Resick et al., 2011; Göçen, 2021). Forsyth et al. (2008) conducted a meta-analysis of more than 80 studies based on relativism and absolutism/idealism. The results showed that Eastern cultures are more likely to believe in God, which explains the high level of absolutism in those cultures. This explains that ethical values in Eastern cultures are derived from religion/spirituality. Spirituality is the basis through which leaders perceive their ethical behavior. The rationale of the facts mentioned above indicates the need to develop a multidimensional ethical leadership scale in the Asian context.

The most important theoretical contribution of this study is developing and validating the person-driven ethical leadership themes from an Eastern perspective. The aim of this study was to fulfill the gap by analyzing the personal characteristics of ethical leadership as suggested by Den Hartog (2015) and Wang et al. (2021). Researchers use a person-driven unidimensional scale that leaves the gap in the literature to explore what characteristics significantly define ethical leadership when used as a composite construct (Yurtkoru et al., 2018; Peng and Kim, 2020). Ethical leader norms and expectations in Eastern societies are different from the Western countries as they are highly rooted in spiritual aspects of everyday life. Therefore, it has been suggested that ethical leadership should be explored by considering the spiritual components (Qasim et al., 2021; Zaim et al., 2021). This study contributes to a mixed methodology using parallel analysis of qualitative and quantitative data strands concurrently. The quality of both qualitative and quantitative data strands from the data collection phase to the data interpretation is fairly justified in this study. This study uses a “Separate approach” of the relational dimension as we are analyzing the qualitative and quantitative data sets independently and are finally merged (Moseholm and Fetters, 2017).

Another worthwhile contribution of this study is can be highlighted by relating it with three dimensions of data integration analytics suggested by Moseholm and Fetters (2017), which are relational, methodological, and dimensional levels. In this study, we are using a “Separate approach” of relational dimension as we are independently analyzing the two strands of data (i.e., qualitative and quantitative) independent of each other and finally merged for the conclusion (Alrawashdeh et al., 2021; Leblebici and Türkan, 2021).

As Forsyth et al. (2008) explained the Ethical positioning theory, the ethics of Western cultures vary from Eastern cultures. The Eastern societies Ethical positioning is based on idealism which stems out from spirituality, whereas, western societies’ ethical positioning is based on relativism, which stems out from promoting ethics on higher cognitive ground philosophy that is rationalizing ethics as a right thing to do. All the above-mentioned points rationalize the need for the development of a multidimensional ethical leadership scale in the eastern scenario. The prior literature review suggested that current ethical leadership scales are based on the western relativism perspective. There is a need to investigate the cultural-driven attributes of ethical leadership, specifically from the Eastern cultural perspective. Mixed method or data triangulation is the integration of quantitative and qualitative research. It was evolved in the mid-1980s, so this method is relatively new, and it has its weaknesses and bias like all other methods. By using both quantitative and qualitative data, the weaknesses of both methods can be neutralized (Creswell, 2014, 2015). The reason for using the triangulation method in this study is to understand the ethical leadership from the eastern (Asian) contextual perspective as it is the most prevalent gap in the literature (Eisenbeiß and Giessner, 2012; Eisenbeiß and Brodbeck, 2014; Ko et al., 2017; Kimura and Nishikawa, 2018; Zaim et al., 2021). In addition, Magalhães et al. (2019) also pointed out that future ethical leadership scales must be based upon multisource research samples. However, Shakeel et al. (2019), in their meta-analysis on ethical leadership, emphasized that future research focused on using alternative method designs other than surveys for better understanding and insight regarding the complete picture of the phenomenon under study (Myers, 2010). This study uses the convergent parallel mixed method for initial item generation in scale development, where the investigator collects qualitative and quantitative data simultaneously and then integrates information from both sources (quantitative and qualitative) for interpretation and development of initial survey items, as shown in Table 1.

**TABLE 1:** Methodological design procedure.

Phase 1	Deductive method	Purpose: themes classification from existing established literature Procedure deductive: Systematic literature review on person-driven characteristics of an ethical leader Nine themes/characteristics frequently prevalent in literature
Phase 2	Inductive method	Purpose: identification and addition of new themes in ethical leadership style in eastern perspective Procedure: Self-administered open-ended questionnaire Demographic respondents seven corporate and seven academic experts The theme generation approach was based on Miles and Huberman (1994) method Survey technique for inductive approach Population and sample Data interpretation and coding Instrumental approach Semantic validity
Phase 3	Deductive + inductive	Purpose: extraction of themes from data triangulation method. Joint display and convergence of deductive + inductive themes Procedure: Semantic validity accumulation record of repetitive corporate and academic responses Convergent parallel mix method 13 themes extraction using qualitative + quantitative technique

## Phase 1: Deductive Approach for Theme Extraction

A deductive approach is used to identify and logically classify themes present in existing literature (Hinkin, 1995). It demands a prior understanding of the context in which the particular phenomenon is addressed. This study emphasizes the person-driven antecedents and characteristics of ethical leadership (Hunt, 1991). The themes/constructs were identified that were repetitively occurring in exhaustive systematic literature reviews (Treviño et al., 2000, 2003; Brown and Treviño, 2006; Resick et al., 2011; Eisenbeiß and Giessner, 2012; Eisenbeiß and Brodbeck, 2014; Ko et al., 2017; Kimura and Nishikawa, 2018; Magalhães et al., 2019) and consistently prevailing in different scales on ethical leadership were identified (Brown et al., 2005; Weaver et al., 2005; Resick et al., 2006; Riggio et al., 2010; Kalshoven et al., 2011b; Moorman et al., 2012; Yukl et al., 2013; Langlois et al., 2014; Zhu et al., 2017; Filho et al., 2019; Zappalà and Toscano, 2020).

### Honesty and Integrity

Treviño et al. (2000) reported that traits such as trustworthiness and honesty contribute to one aspect that respondent called “moral person.” It has been explained by Kalshoven et al. (2011a), Yukl et al. (2013), and Zappalà and Toscano (2020) that honesty and integrity are doing the real thing for the right reasons in the correct way. Reliable, ethical leaders are those who possess the attributes of honesty and integrity. Such leaders keep their promises and behave more consistently and predictably.

### Fairness

Fairness, as suggested by Treviño et al. (2003), Brown et al. (2005), De Hoogh and Den Hartog (2008), Kalshoven et al. (2011a), Resick et al. (2011), Yukl et al. (2013), Ko et al. (2017), and Filho et al. (2019), is seen as an important form of ethical leader behavior and explained as making fair and just-based objective decisions and not discriminate against others. So, ethical leaders treat others fairly by making principled and fair choices, not practicing favoritism, and taking responsibility for their actions.

### Accountability

Accountability, as explained by Conger (1989) and Konczak et al. (2000) and suggested by Treviño et al. (2003), Resick et al. (2006), Resick et al. (2011), and Zappalà and Toscano (2020), includes complying with laws and regulations, holding others accountable, and making them responsible. It is about promoting ethical principles in the organization and holding the people accountable for their performance, which they can control. This makes accountability a mechanism by which responsibility for outcomes is given to individuals and teams.

### People Orientation and Consideration

People orientation and consideration, as coined by Kanungo and Conger (1993) and suggested by Treviño et al. (2003), Resick et al. (2006), Resick et al. (2011), Kalshoven et al. (2011a), Filho et al. (2019), and Zappalà and Toscano (2020), is the dimension of ethical leadership. It means showing respect for others and treating them with dignity and respect. Ethical leaders should be approachable, good-natured, empathetic and understanding, and helpful in protecting and developing their staff or having true concern for people. The people-orientation component in ethical leadership reflects genuinely caring about, respecting, and supporting subordinates and ensuring as possibly as they can that their staff needs are adequately met.

### Moral Identity

Moral identity was coined by Aquino and Reed (2002), suggested by Den Hartog (2015), and further by Magalhães et al. (2019). Mayer et al. (2012) defined it as a self-schema, self-regularity mechanism based on personal moral traits embedded in the leader’s self-concept and behavior.

### Internal Locus of Control

Internal locus of control, as developed by Rotter (1966), and proposed by Trevino (1986) and Brown and Treviño (2006), and also emphasized by Ko et al. (2017), is the dimension of ethical leadership. It is perceived that individuals with an internal locus of control have greater control over themselves. As a result, they would behave more ethically because they are more likely to perceive the connection between their behavior and the outcomes produced by that behavior. As a result, they are more likely to take responsibility for the outcomes of their actions. Thus, internal locus of control leaders takes more responsibility for the outcomes of their actions on other people.

### Role Modeling

Role modeling, as explained by Weaver et al. (2005) and further used by Zhu et al. (2017), is considered as modeling behaviors that are (1) interpersonal based on care, concern, compassion, supporting, hardworking, helping, and taking responsibility; (2) self-projection of ethical action (setting high standards of self based on honesty, humility, trustworthiness, and integrity, self-sacrifice, and taking responsibility for personal failures and ethical consistency); (3) fairness with others (explaining and fair treatment to others with respect); and (4) articulation of ethical standards (demonstrating and communicating high standards, ensuring accountability, putting ethics in priority, long-term oriented, and having a multi-perspective).

### Personality Traits

Personality traits, as presented by Hendriks et al. (1999) and suggested by Kalshoven et al. (2011a) and Den Hartog (2015) and further by Magalhães et al. (2019), which are classified into conscientiousness traits, such as dutiful, thorough, responsible, organized, and agreeable traits, such as caring, kind, trustworthy, and empathetic, should be considered in defining ethical leader traits.

### Moral Reasoning/Ethical Critique

Moral reasoning/ethical critique, as developed by Kohlberg (1972, 1981) and named as post-conventional level by Starratt (1991) and as ethical critique proposed by Brown and Treviño (2006), Jordan et al. (2013), Langlois et al. (2014), and Den Hartog (2015), is one of the aspects of ethical leadership. It states that “basic leader stance is ethical for they are dealing with questions of social justice and human dignity, although not with individual choices.” These leaders were deeply concerned about issues of social justice that they witnessed and were ready to take action to preserve equity in their organizations.

## Phase 2: Inductive Approach for Item Generation

Following the deductive approach, an inductive approach was used, involving the researchers inquiring the respondents to explain the constituents defining the construct under study (Hinkin, 1995). Qualitative research is based upon human experience in the naturalistic surroundings, personal understanding, and social process. Thus, the inductive approaches in social sciences work in open systems rather than closed ones (Glaser and Strauss, 1967; Miles and Huberman, 1994).

This inductive approach for item generation is based on the following procedures:

I Survey technique for an inductive approach
II Population and sample (Study 1)
III Data interpretation and coding
IV Instrumental approach
V Semantic validity

### Survey Technique for an Inductive Approach

Questionnaires and interviews are some of the most accepted and commonly used techniques in survey research for data collection in social science research (Churchill and Iacobucci, 2006). The survey method selected for current research is a self-administered open-ended questionnaire, which is deemed appropriate according to Bryman and Bell (2015).

Rowley (2014) explained them as documents that can include a single to series of open questions that invited the respondents to their answer without having any direct face-to-face or even remote interaction with the researcher. This method is financially and methodologically appealing and provides convenience to the respondents to think and state their answers in a relaxed environment (Sax et al., 2003). Moreover, questionnaires give the respondents complete anonymity, which ensures the reliability and accuracy of information obtained from the respondents (Creswell, 2014).

Another reason for selecting this technique in this study is explained by Friborg and Rosenvinge (2013), who showed that an open-ended questionnaire provided more in-depth information than close-ended questions. An open-ended questionnaire contributes a lot, especially in theory construction and addition. The purpose of conducting this inductive research is to gain an in-depth context-based eastern perspective on the definition of ethical leadership. Usually, an open-ended questionnaire inquires for what, who, where, when, and why.

This study was based on four demographic questions (asking organization name, department, designation, and years of experience in their field), the details are shown in Table 2, followed by a single open-ended question that inquired from the respondent to “describe according to their view an ethical leader should possess what characteristics?”

**TABLE 2:** Demographic analysis.

Demographic characteristics	Categories	Frequency	% Age
Gender	Male	09	64
	Female	05	36
Age	31–40	08	57
	41–50	06	43
Education	Masters	04	28
	MS/M.Phil.	03	22
	Ph.D.	07	50
Marital status	Single	05	36
	Married	09	64
Organization type	Public	05	36
	Private	09	64
Working experience	5–10	02	14
	11–15	07	50
	>15	05	36

### Population and Sample (Study 1)

The sampling design of this qualitative research is of a single stage as the researcher has access to the population and can directly sample the target (Creswell, 2014).

In qualitative research, the idea is to purposefully and carefully choose the participants or the sites (documents or the visual material), which will aid the researcher in understanding the research question or the problem. Similarly, qualitative research does not require any specific sampling technique or selection of a large group of participants, unlike quantitative research. Miles and Huberman (1994) discussed four aspects to be covered: the setting that is where the research will take place, the actors mean the respondents whose response will be collected, the events mean what the respondents will be asked, and the process through which the evolving nature of data will be captured and interpreted.

Apart from the sampling technique, there lies another ambiguity in qualitative research. Researchers provide no specific answer to address how many participants and sites are involved in the study. However, Creswell (2013) explained that sample size depends upon the qualitative design used. Many qualitative research studies used narrative research by including just one or two individuals, whereas phenomenology typically used ranges from 3 to 10. While in grounded theory, probably 20–30 respondents are required, and ethnography examines four to five cases. This is not a generalized approach to identifying the sample size. Another approach and the more viable idea of saturation presented by Charmaz (2006) derived from the grounded theory as he explained that the researcher should stop collecting the data when the themes or the categories started getting saturated, which means that when gathering of new data do not any longer sparks the new insights and reveals new themes, at that stage data collection should be stopped.

Keeping in view the above points, the open-ended questionnaire was circulated through Email among 25 working professionals in the middle management level having more than 10 years of working experience either in the corporate sector or academia. Out of which 14 respondents replied. The informants included seven from the corporate sector (Supplementary Annexure II) and seven from higher education (Supplementary Annexure III).

The reason for including the corporate and academia respondents was to generalize the scale validity in the service sector. Similarly, the reason for selecting middle management with 10 years of experience was to target informants who have worked not only in subordination but also as supervisors or in leadership roles in their organizations so that they could comprehend their perspective and envisioned ethical leadership from the perspective of both roles.

### Data Interpretation and Coding

The responses to the open-ended questions are usually provided descriptively. Respondents sometimes present their descriptions in the form of listings that demonstrate their knowledge regarding the phenomenon, explanation, or sometimes motivation. These descriptions are based on facts, but sometimes their attitudes or evaluations are also represented in their feedback. Similarly, the arguments sometimes are mentioned explicitly, and conversely, sometimes the researcher has to read and analyze between the lines due to the subjectivity of data. To get specific, meaningful, and interpretable data from the open-ended questionnaire, the key lies in the formulation of the right and relevant question. They are keeping in view that the formulation of the question should be neutral and inviting to answer. Similarly, questions should be specific, short, and contain the right questioning word (Popping, 2015).

Roth (1971) experienced the issues with the responses to the open-ended questions that they are sometimes not completely clear and often contained very ambiguous words and phrases. Most of the time, they have grammatical issues and are poorly worded. Conversely, Geer (1988) stated that most respondents do communicate an adequate and sufficient answer.

Krosnick (1999) explained three steps that the respondents in a survey confront. First, they interpret the question as per their understanding and deduce the intent of the question. Second, they must retrieve from their cognitive process the relevant information and amalgamate that information or understanding into a particular judgment. Finally, they translate their judgment into their responses.

In the current scenario, some of the respondents specifically stated the characteristics of an ethical leader. Some respondents explained the characteristics that an ethical leader should possess. Altogether, the respondents clearly understood the question as they communicated adequate answers to define the characteristics of an ethical leader.

The open-ended question contains a set of codes. Coding means the process through which the raw data are transformed systematically and combined into themes or units containing the detailed description of the relevant matter characteristics (Holsti, 1969). The respondents, based on their understanding and judgment, give a response to the particular question. The researcher will categorize their response into the relevant theme or code.

In this study, data coding was made by recording and combining the phrases or words similar in meaning into similar themes. In this step, the data were systematically coded into 13 themes that are listed as follows:

1. Agreeable/respect/respectful/well-mannered/humble
2. Conscientious/conformity/dutiful/dedicated/devotion with organizational values
3. Fairness/justice/unbiased/transparent
4. Honesty and integrity/truthfulness/not betraying
5. Team oriented/collective good/common good
6. Accountability/no tolerance for values
7. Humane/people centric/positive/empathy/forgiving/tolerance/approachable/caring
8. Locus of control/responsible
9. Role modeling/credibility/trustworthy/trust building/upright
10. Altruism/selfless/sincerity
11. Give credit/confident/secure/appreciative/skill building/encourage initiative
12. Ethical critique/brave/strong will power/understanding/flexibility/analytic ability
13. Wisdom/faith/charisma/vision/preaching/personal knowledge/mission-oriented/sense of connecting with the purpose of life

### Instrumental Approach

The instrumental approach involves the researcher’s theory to interpret the texts according to it. In this approach, the researchers develop the themes’ dictionaries that reflect how these themes will be interpreted before data coding. Here, the researchers must use sympathetic understanding for encoding the text according to their sources’ meaning. This representational way requires the researcher to manually code the fragmented text into the assigned theme so that the coder could capture the latent meaning prevailing in the text according to the theme, which cannot be captured if a computer program is being used (Shapiro and Markoff, 1997).

In this study, the instrumental approach was followed, because, through the deductive method, the themes were already generated from past literature that explained the concept of ethical leadership. However, by following the inductive method, some new themes also emerged from the respondent’s descriptions and feedback, which were not considered part of ethical leadership in previous literature in the Western context, such as altruism, collective good, encouragement, and transcendence spirituality. In contrast, some researchers (Resick et al., 2006; Eisenbeiss, 2012) in cross-culture context highlighted and suggested them to be part of ethical leadership in eastern cultures.

### Semantic Validity

After the respondent’s response or answer is coded down by a coder (or the computer program) into themes, it leads toward semantic validity. A meaning category, theme, or the code semantic validity means that the researcher who already has familiarity with the texts underneath the study has the consensus among the categorization of the phrases or words collected through responses and combined or recorded into a particular category or theme do reflect and quantify that categories or theme meaning (Krippendorff, 2012).

The assurance of the semantic validity is more required than the face validity, which is based on checking similarities among words and phrases with themes but extended toward making the justifiable inferences relied upon the collection of reliable and valid data (Cavanagh, 1997).

In this study, semantic validity was performed (Table 3) by carefully checking that words or phrases categorized in themes defining ethical leadership properly and making tallies that how many times the similar responses by corporate and academia occurred in the particular theme defining the ethical leadership.

**TABLE 3:** Themes emerging from a questionnaire response.

	Themes for the characteristics of ethical leadership	Corporate and academia response
1.	Agreeable/respect/respectful/manner-full/humble	C1, C5, A1, C2
2.	Conscientious/conformity/dutiful/dedicated/devotion with organizational values	C2, C3, C4, C5, A1, A4, A6
3.	Fairness/justice/unbiased/transparent	C1, C2, C5, C6, C7, A1, A2, A6
4.	Honesty and integrity/truthfulness/not betraying	C1, C2, C5, C6, C7, A1, A2, A6, A7, A8
5.	Team oriented/collective good/common good	C1, C2, C3, C5, C7, A1, A2, A3, A4, A7
6.	Accountability/no tolerance for values	C1, C6, A2, A3
7.	Humane/people centric/positive/empathy/forgiving/tolerance/approachable/caring	C1, C2, C3, C5, C6, A1, A2, A3, A6, A7, A8
8.	Locus of control/responsible	C8, A7, A8
9.	Role modeling/credibility/trustworthy/trust building/upright	C3, C4, C5, C7, A1, A2, A3, A4, A7
10.	Altruism/selfless/sincerity	C3, C4, C7, A4
11.	Give credit/confident/secure/appreciative/skill building/encourage initiative	C3, C4, C5, C7, A1, A2, A3, A4, A7, A8
12.	Ethical critique/brave/strong will power/understanding/flexibility/analytic ability	C3, C5, C6, C7, A3, A4, A6, A7
13.	Wisdom/faith/charisma/vision/preaching/personal knowledge/mission oriented/sense of connecting with purpose of life	C3, C4, C6, C8, A1, A3, A5, A6, A7

*A1, A2, A3, A4, A5, A6, A7, A8 for academic responses.*

*C1, C2, C3, C4, C5, C6, C7 for corporate responses.*

## Phase 3: Convergent Parallel Mixed Method

For data triangulation and analysis, the convergent mixed method design was applied in this study to merge or converge the themes/dimensions extracted from deductive and inductive approaches. In this design, the two databases (quantitative and qualitative) are separately analyzed and then merged. For merging the two databases, several ways are identified by Creswell (2014).

This study follows the side-by-side comparison approach. In this approach, the researcher presented the comparison within the discussion by first explaining one set of the findings; in this study, themes/dimensions were first extracted using the deductive method, followed by the themes/dimension extracted using the inductive method. The final procedure was to merge and to integrate these two forms of data into the graphic form or the table form, which is also known as the joint display of the data, and this can be displayed into many different forms (Creswell et al., 2011; Creswell, 2014). Using mixed-method design in this study was to identify the characteristics-driven themes/dimensions to develop the context-based ethical leadership questionnaire. So, based on convergence, 13 dimensions/themes were identified in which 9 themes (agreeable, conscientious, fairness, honesty and integrity, accountability, people-centric, internal locus of control, role modeling, and ethical critique) were supported by both deductive and inductive methods.

In contrast, four additional context-based themes (i.e., altruism, collective good, encouragement, and spiritual transcendence) emerged from the inductive method. However, among these new themes, Resick et al. (2006) previously identified three themes/dimensions (i.e., altruism, collective good, and encouragement) of ethical leadership were also identified. However, they were not recommended and used further by any other researcher. In contrast, the spiritual or transcendence-based dimension is the new context-relevant dimension that emerged from the inductive method.

Table 4 shows all deduction-based nine dimensions with the inclusion of four new dimensions were merged into the table, where the themes array in the horizontal axis and the categorical variables (i.e., quantitative and qualitative) on the vertical axis (Creswell, 2014) were considered to be a valuable addition to this study to define the context-based characteristics of an ethical leader.

**TABLE 4:** Themes emerging from triangulation.

	Emerging themes	Quantitative	Qualitative
1.	Agreeable/respect/respectful/manner-full/humble	✓	✓
2.	Conscientious/conformity/dutiful/dedicated/devotion with organizational values	✓	✓
3.	Fairness/justice/unbiased/transparent	✓	✓
4.	Honesty and integrity/truthfulness/not betraying	✓	✓
5.	Team oriented/collective good/common good		✓
6.	Accountability/no tolerance for values	✓	✓
7.	Humane/people centric/positive/empathy/forgiving/tolerance/approachable/caring	✓	✓
8.	Internal locus of control/responsible	✓	✓
9.	Role modeling/credibility/trustworthy/trust building/upright	✓	✓
10.	Altruism/selfless/sincerity		✓
11.	Give credit/confident/secure/appreciative/skill building/encourage initiative		✓
12	Ethical critique/brave/strong will power/understanding/flexibility/analytic ability	✓	✓
13.	Wisdom/faith/charisma/vision/preaching/personal knowledge/mission oriented/sense of connecting with purpose of life		✓

### Contribution to Mixed Methodology

This study contributes to a mixed methodology by using convergent parallel track analysis of two data (i.e., qualitative and quantitative) strands simultaneously. The novelty of this methodology is highlighted by Hatta et al. (2018) as they emphasized that it is very difficult and complicated to maintain separate strands of data either in the collection phase or in the analysis phase. Usually, variations occur, and data cross-over at some time between these phases because of overlapping aims and reasoning styles in research; this makes complexities in pursuing concurrent methodologies independently. Conversely, this study fairly justifies the quality of independence of both data (i.e., qualitative and quantitative) strands independently from the point of data collection to data interpretation using a two-lens framework for deeper insight and finally merging (qualitative + quantitative) strands together to gain a superior perspective on ethical leadership domain.

Another worthwhile contribution of this study can be highlighted by relating it with the three dimensions of data integration analytics explained by Moseholm and Fetters (2017), namely, relational, methodological, and dimensional levels. In this study, we are using a “Separate approach” of relational dimension as we are independently analyzing the two strands of data (i.e., qualitative and quantitative) independent of each other and finally merged them together for the conclusion. Second, in the methodological dimension, this study uses the “Equal status approach,” where the strategy of this research was to equivalently understand both (qualitative and quantitative) epistemologies and extract deeper insights from them (Kalayci et al., 2021). Finally, this study uses the “simultaneous bidirectional dimension” approach, where we simultaneously and independently collected qualitative (literature review)-based data as well as the quantitative (open-ended questionnaire driven) data responses and separately analyzed them and then merged them together for concluding convergence and divergent interpretations and extractions from data.

## Conclusion

This study follows the parallel comparison approach by first extracting the themes of personal-driven ethical leadership characteristics through the deductive method followed by the inductive technique. Furthermore, the final procedure was based on integrating and merging the two sets of data (deductive and inductive) in a joint display. The purpose of using mixed-method design specifically in this study was to explore the eastern context-based characteristics/themes of the personality-driven, ethical leadership domain through the convergence of 13 themes. Among them, nine themes were based on both inductive and deductive techniques, whereas this study identified four additional context-driven themes through the inductive method (i.e., altruism, encouragement, collective good, and spiritual transcendence) in the domain of ethical leadership from the eastern perspective. However, Resick et al. (2006) identified the prevalence of three themes in Eastern societies having collectivist cultures, namely, altruism, collective good, and encouragement, and explained that the ethical leaders are perceived as fatherly figures and tend to exhibit their focus on collective good, acknowledge individual contributions, and sincere in providing support and feedback to their employees, but these themes remain underexplored and were not represented in the scales. In addition, the theme of spiritual transcendence also emerged from the inductive method, and Eisenbeiß and Giessner (2012) highlighted in their study that the ethical leadership of Eastern cultures projects religious/spiritual orientation due to their ethical positioning based on the absolutism paradigm; still, this theme remains untapped and underresearched as the characteristics of ethical leadership. So, this study identified these four themes as the study gap in the existing ethical leadership scales. These findings will be considered a valuable addition in the investigation of context-based ethical leadership characteristics.

### Theoretical Implications

This study deconstructs the reality and reconstructs it according to the cultural realities of our respective societies. Ethical leadership literature is based on Western culture and completely ignores the Eastern cultural realities. This study explores the ethical leadership phenomenon from the lens of ethical positioning theory, which explains that ethics defined in Western cultures are different from the ethics perceived in Eastern cultures. The ethical positioning of the eastern cultures is based on idealistic cultural values, whereas the western societies rely on relativism based ethical positioning. In the line with the above theoretical arguments, this study investigates the ethical positioning of a leader within the collectivist, high power distance, and high uncertainty avoidance culture.

### Practical Implications

This study is based on the realism approach that is based on the eastern perspective of ethical leadership. As our culture is deeply rooted in spiritual and social values that have been ignored by the west, norms and expectations of employees in our culture are different as they are embedded in spiritual aspects. This study explored the characteristics of ethical leadership from the cultural lens that will benefit managers and practitioners to cater to the needs of their followers such as enhancing their innovative work behavior for gaining competitive advantage. It will also help managers to overcome the organization’s dehumanization and will increase the job commitment and performance of the employees. Managers need to be honest, truthful, and fair for developing an ethical identity that will reduce the prevalence of negative behaviors within the organization.

## Data Availability Statement

The raw data supporting the conclusions of this article will be made available by the authors, without undue reservation.

## Ethics Statement

The studies involving human participants were reviewed and approved by the Research Ethics Approval Committee, Faculty of Management Sciences, National University of Modern Languages, Islamabad. The patients/participants provided their written informed consent to participate in this study.

## Author Contributions

All authors listed have made a substantial, direct, and intellectual contribution to the work, and approved it for publication.

## Conflict of Interest

The authors declare that the research was conducted in the absence of any commercial or financial relationships that could be construed as a potential conflict of interest.

## Publisher’s Note

All claims expressed in this article are solely those of the authors and do not necessarily represent those of their affiliated organizations, or those of the publisher, the editors and the reviewers. Any product that may be evaluated in this article, or claim that may be made by its manufacturer, is not guaranteed or endorsed by the publisher.
