# Supplementary material for: Characteristics of Ethical Leadership: Themes Identification Through Convergent Parallel Mixed Method Design From the Pakistan Context
Source: Front Psychol. 2021 Dec 17;12:787796. doi: 10.3389/fpsyg.2021.787796 (PMC8719645; doi:10.3389/fpsyg.2021.787796)
Supplement: Supplementary file 1 [file Data_Sheet_1.pdf]

Annexure I

**Subject:**	**Research Work**

Dear Sir/Madam

We are conducting academic research on Titled**: Characteristics of Ethical Leadership themes identification through Convergent Parallel Mixed Method Designs**

This interview is designed for academic and professional members who are in middle management and/supervisors themselves. Difficult words are elaborated within brackets to help respondents in understanding the question clearly.
Kindly provide your best possible support in terms of data collection through the questionnaire.

Your support will give great impetus and encouragement in the completion of research in time. I hope that you will extend your cooperation in all the best possible ways.
I will be very grateful for your support in this regard.
With best regards,

Dr HINA SHAHAB, Dr Hafsa Zahoor and Dr Rabia Imran
Corresponding Email: hina.shahab@gmail.com

**Organization Name:**		**Years of experience in your field:**
**Designation:**		**Department:**
**Question:**	**Describe according to your view an ethical leader should possess what characteristics**	

Thank you for your time and valuable contribution!

Annexure II Corporate Response

**Name:** Sehrish Sindhu		**Organization:** Power99 Foundation
**Designation:** Manager Finance		**Department:** Finance
**Question:**	**Describe according to yours view an ethical leader should possess what characteristics?**	

According to me, an ethical leader should have below mentioned characteristics;

1. Humane
2. Encourages initiative
3. Respect others
4. Justice
5. Honest
6. Focus on teambuilding
7. No tolerance for ethical violations
8. Radiate Positivity
9. Wisdom
10. Spirituality

**Name: Jamal Abbas**		**Organization:Chez soi**
**Designation: Manager**		**Department: Admin**
**Question:**	**Describe according to your view an ethical leader should possess what characteristics**	
An ethical leader is always fair. They have no favorites and treat everyone equally. Under an ethical leader, no employee has any reason to fear biased treatment based on gender, ethnicity, nationality, or any other factor.		
Ethical leaders emphasize being kind and acting in a manner that is always beneficial to the team.		
In ethical leadership, all decisions are first checked to ensure that they are in accordance with the overall organizational values. Only those decisions that meet this criterion are implemented.		
Ethical leadership is not just about talking the talk. This type of leader also walks the walk. The high expectations that an ethical leader has of employees are also applicable on the individual level. Leaders expect others to do the right thing by leading by example.		

Ethical leaders speak to us about our identity, what we are and what we *can* do the cast of mind that lets you practice *your* principles consistently.

**Name:**<br>**Muazzam Rasool**	**Organization:**<br>**Capital Group of Industries**
**Designation: manager operations**	**Department: logistics div**
**Question:**	**Describe according to your view an ethical leader should possess what characteristics**
**A 1-**A good leader must have intelligence and imagination to create a compelling vision of the future and bring those who can deliver it with them.	
**A 2-**A good leader must also be trustworthy, display unshakeable integrity, be action-oriented, and be resilient in the face of setbacks while treating people with respect, not as mere units of production.	
**3-**They are seen to act from their own well-developed set of ethical principles, setting a consistently good example for others to follow	
**4-**Ethical leaders are strong on selfless service in the interests of the greater good	
**The 5-**Their door is always open	
**6-**Ethical Leaders allow and appreciate their subordinates call you out, disagree with you, challenge your judgment; all of this calls for great understanding and tolerance	
**7-**The ethical leader accepts that they are either directly or indirectly responsible for everything that happens in the organization. They understand that blame-shifting and finger-pointing is a failure of leadership, as we saw in the VW fiasco when the CEO sought to blame the engineers and technicians. The ethical leader does not resort to the "plausible deniability" defence.	

**Name: Syed Ali Abbas Baqir**	**Organization: Baqir Travel & Tours**
**Designation: Director**	**Department: N.A.**
**Question:**	**Describe according to your view an ethical leader should possess what characteristics**
**Well it's tough to give a concise answer but I do believe that a true leader should lead from**	
**the front and be active and energetic, also be able to adapt to certain problems as they**	
**arise within the organization and be able to suggest a solution for it.**	
**As i said earlier, a true or ethical leader should be someone who should act first and lead**	
**by example, for his subordinates to see and believe in him/her, so it has to be;**	
**Practice what you preach! Not the opposite of it!**	
**Besides this, an ethical leader, I believe, should have a little charisma as well for the people**	
**to be inspired by him/her.**	
**Similarly, an ethical leader should be able to express himself or herself and be able to**	
**lead by example.**	
**The sincerity of the ethical leader is also of prime importance.**	
**I believe that's all what I can think of for now!**	
**Thank You for your questionnaire & asking me to be a part of it!**	

**Name:faizan**		**Organization:SNL**
**Designation:**		**Department:**
**Question:**	**Describe according to your view an ethical leader should posses what characteristics**	
1 Justice: A person should have one quality that is justice in every meters of life.		
2 Respect others: (Respect every one still if anyone is too junior)		
3. Honesty (To promote any one to reward, To divided work load)		
4. Humane (Must have sense that people are not report.		
5. Together we stand divided we fall (Must be a team builder)		
6. Value driven decision-making		
7. Encourages initiative		
8. Leadership by example		
9. Should plan awareness programs for subordinates.		
10. Must have tolerance		

Name: Rehan Khan		Organization: USAID citizens Voice Project
Designation: Manager		Department: Communications & External Relations
Question:	Describe according to your view an ethical leader should possess what characteristics	
An ethical leader, in my opinion should possess the following characterizes.		
a) Transparent- He should not give undue favors and should treat everyone equally.		
b) Accountable- He should hold himself accountable when he makes a mistake and be honest.		
c) Approachable- He should inspire trust within his team so they can approach him in confidence.		
d) Empathetic – He should be aware of the limitations of each team member and not penalize them at every turn. Thus, he should genuinely respect them.		
e) Humble – He should not be arrogant and should keep himself grounded. Thus, he should not exercise his managerial powers (unless needed) to force his team to act according to his wishes.		
f) Patient – He should have a well balanced personality where he thinks and assesses a situation patiently to decide for the overall well-being of his team.		
g) Strategic- He should have foresight and envision a stronger future for every member of his team.		
h) Brave – He should be willing to take a stand to defend his team when needed. Similalry, he should be brave to confront his team in case anyone is not performing well.		
i) Confident- He should be sure of himself and only take action once he has thought about things in a systematic way.		
j) He should not be insecure as a professional or as a person.		
k) Should give due credit to his team when it is due. He should duly appraise them.		
l) Have the capacity to develop on-to-one personal relationships with them.		

m) Should be an upright individual who inspires trust within his subordinates
n) Should utilize and develop the skill set of his team and help them become better professionals.<br>o) Professional and result oriented- an empathetic leader should set quality standards and inform his team about them. So the team knows how to work to meet the quality standards.

**Name: Rizwan Ali**		**Organization: NUML**
**Designation: SO to DG, NUML**		**Department: NUML**
**Question:**	**Describe according to your view an ethical leader should posses what characteristics**	
**Integrity**		
**Honesty**		
**Encourage Initiative**		
**Exemplary Character**		
**Humane**		
**Encourage Team work**		
**Good Will Power**		
**Take stand on decisions**		
**Rational decision making**		
**Do Justice**		
**Unselfishness**		

Annexure III Academic Response

**Name:Sehar Zulfiqar**	**Organization: NUML**
**Designation: Assistant Professor**	**Department:FMS**
**Question:**	**Describe according to your view an ethical leader should possess what characteristics**
**Persuasive …….leading by example ethical standards**	
**Honest…….**	
**Will do justice ….even that will put him into trouble**	
**Will stick to follow morals and ethical standards even it will be hard to follow**	
**Will continuously give reminders through discussions what is right and what is wrong according to social, organizational and religious standards**	
**Respect others …..also respect the values and beliefs of them… regarding their moral standards**	
**Will make fair decisions**	
**Will have ethical considerations in making decisions**	

**Name: iffat rasul**	**Organization: Hamdard University**
**Designation: lecturer**	**Department: MS**
**Question:**	**Describe according to your view an ethical leader should possess what characteristics**
**Ethics is usually associated with the social exchange. Ethical leadership tens to motivate the employees through organizational trust. In most of the researches on social exchange, trust is ignored for developing any behavior. Therefore, leaders should keep his words because the employees who find their leaders keeping their promises are considered trust worthy. He should be loyal and honest.**	
**He should respect the employees and should not go personal while making any organizational decision**	
**He should treat employees humanely and should kind natured.**	
**He should not compromise on ethical values**	
**He should open the doors of communication and give value to the voice of employees.**	
**He should be transformation for appreciating the innovative efforts of employees**	
**He should be able o infuse team building sprit among the employess.**	

**Name: Fatima Ashraf**	**Organization: NUML Islamabad**

**Designation: Lecturer**		**Department: Management Sciences**
**Question:**	**Describe according to your view an ethical leader should possess what characteristics**	
1. Empathize with colleagues; understand their personal, educational, physical, emotional restraints and enhance their work performance understanding these.		
2. Remind workers of moral and codes of conduct in a 'slight' way.		
3. See employees beyond their individual, flawed personalities, but be perfectly capable of enhancing their work performance keeping in view these.		
4. Set personal examples of truthfulness, honesty.		
5. Never be judgmental or disapproving, at least openly, but, as a leader, be capable of minimizing these for work performance.		
6. Be capable of understanding complexities in people and situations.		
7. Be a responsible decision-maker.		
8. Clearly spell the behaviour that the business should not engage in.		
9. Set a work environment where fairness, honesty, empathy is rewarded, quoted.		

**Name: Dr Hina Rehman**		**Organization: NUML**
**Designation: Assistant Professor**		**Department: Management Sciences**
**Question:**	**Describe according to your view an ethical leader should possess what characteristics**	
**If I divide this terminology into two parts, ethical and leader. Then first comes with the word ethical in my point of view, it means doing the duty within the set pattern and according to rules, and a leader is the person who demonstrates this through his or her actions, not words. So ethical leader is the person who acts or perform duty within the spirit of organization rules. The person who maintains integrity and honesty and stake his or her personal interest in favour of the organization interest. Whom the employees of the organization can trust. The employees feel that my leader never betrays us. The leader who respect their employees. The leader who never make a grudge and grievance to any of the employees.**		

**Name: Usman Kemal**		**Organization: IQRA University Islamabad**
**Designation: Assistant Professor**		**Department: Management Sciences**
**Question:**	**Describe according to your view an ethical leader should possess what characteristics**	
**An Ethical Leader should be:**		
**Honest**		
**Decision maker**		
**Initiator**		
**Confident**		
**Integrity**		
**Passionate**		

**Innovator**
**Patience**
**Inspiring**
**Open-minded**
**Positive**
**Good communicator**

<table>
<tr><td colspan="2">Name: Fatima Ashraf</td><td>Organization: NUML Islamabad</td></tr>
<tr><td colspan="2">Designation: Lecturer</td><td>Department: Management Sciences</td></tr>
<tr><td>Question:</td><td colspan="2">Describe according to your view an ethical leader should possess what characteristics</td></tr>
<tr><td colspan="3">1. Empathize with colleagues; understand their personal, educational, physical, emotional restraints and enhance their work performance understanding these.</td></tr>
<tr><td colspan="3">2. Remind workers of morals and codes of conduct in a 'slight' way.</td></tr>
<tr><td colspan="3">3. See employees beyond their individual, flawed personalities, but be perfectly capable of enhancing their work performance keeping in view these.</td></tr>
<tr><td colspan="3">4. Set personal examples of truthfulness, honesty.</td></tr>
<tr><td colspan="3">5. Never be judgmental or disapproving, at least openly, but, as a leader, be capable of minimizing these for work performance.</td></tr>
<tr><td colspan="3">6. Be capable of understanding complexities in people and situations.</td></tr>
<tr><td colspan="3">7. Be a responsible decision-maker.</td></tr>
<tr><td colspan="3">8. Clearly spell the behaviour that the business should not engage in.</td></tr>
<tr><td colspan="3">9. Set a work environment where fairness, honesty, empathy is rewarded, quoted.</td></tr>
</table>

<table>
<tr><td colspan="2">Name

<u>Abdul Rahman</u></td><td>Organization

<u>International Islamic University</u></td></tr>
<tr><td colspan="2">Designation

<u>Business and Soft Skills Instructor</u></td><td>Department

<u>Business Administration and Management (BAM)</u></td></tr>
<tr><td>Question:</td><td colspan="2">Describe according to your view an ethical leader should possess what characteristics</td></tr>
<tr><td colspan="3">I believe an ethical leader should possess the following traits to lead his or her followers effectively.

1. Vision
Unless a leader has the vision to realize, he or she will not be able to convince and guide the followers.

2. Credibility</td></tr>
</table>

If the leader does not establish credibility, the followers will not believe in him. This is achieved through being a role model.

3. **Resilience**
   The leader should have flexibility and patient to look at others' points of view and not perceive this as a threat to his or her power.

4. **Humility**
   Prophet Muhammad (SAW) is a role model. Nelson Mandela is another example. A leader should be humble enough to forgive and forget if he or she desires to move toward achieving goals.
